# The Hepatitis B Virus Interactome: A Comprehensive Overview

**DOI:** 10.3389/fmicb.2021.724877

**Published:** 2021-09-16

**Authors:** Ellen Van Damme, Jolien Vanhove, Bryan Severyn, Lore Verschueren, Frederik Pauwels

**Affiliations:** ^1^Janssen Research & Development, Janssen Pharmaceutical Companies, Beerse, Belgium; ^2^Early Discovery Biology, Charles River Laboratories, Beerse, Belgium; ^3^Janssen Research & Development, Janssen Pharmaceutical Companies, Springhouse, PA, United States

**Keywords:** hepatitis B virus, interactome, viral-host life cycle, cccDNA, HBx, HBc

## Abstract

Despite the availability of a prophylactic vaccine, chronic hepatitis B (CHB) caused by the hepatitis B virus (HBV) is a major health problem affecting an estimated 292 million people globally. Current therapeutic goals are to achieve functional cure characterized by HBsAg seroclearance and the absence of HBV-DNA after treatment cessation. However, at present, functional cure is thought to be complicated due to the presence of covalently closed circular DNA (cccDNA) and integrated HBV-DNA. Even if the episomal cccDNA is silenced or eliminated, it remains unclear how important the high level of HBsAg that is expressed from integrated HBV DNA is for the pathology. To identify therapies that could bring about high rates of functional cure, in-depth knowledge of the virus’ biology is imperative to pinpoint mechanisms for novel therapeutic targets. The viral proteins and the episomal cccDNA are considered integral for the control and maintenance of the HBV life cycle and through direct interaction with the host proteome they help create the most optimal environment for the virus whilst avoiding immune detection. New HBV-host protein interactions are continuously being identified. Unfortunately, a compendium of the most recent information is lacking and an interactome is unavailable. This article provides a comprehensive review of the virus-host relationship from viral entry to release, as well as an interactome of cccDNA, HBc, and HBx.

## Introduction

Hepatitis B virus (HBV) is a member of the *Hepadnaviridae* family which is transmitted via bodily fluids as well as by vertical transmission (Davis et al., 1989; Schweitzer et al., 2015). The outcome of HBV infection is determined by multiple host and viral factors, and determines whether the infection will be acute, chronic, or occult (Fanning et al., 2019). Despite the availability of a prophylactic vaccine and potent antiviral treatments, chronic hepatitis B (CHB) infection affects 292 million individuals worldwide (Lazarus et al., 2018). The current standard of care is treatment with nucleos(t)ide analogs (NUCs) (i.e., lamivudine, adefovir, entecavir, telbivudine, and tenofovir), that inhibit the HBV polymerase reverse transcription (Liang et al., 2015). These therapies lead to suppression of viral replication, visible by a decrease in viral load, the normalization of serum alanine transaminase and improvement of liver histology (Bitton Alaluf and Shlomai, 2016). However, even prolonged treatment with NUCs rarely results (<10%) in functional cure of CHB and most often leads to virological relapse after treatment cessation (Liang et al., 2015; Kim, 2018).

Also pegylated interferon alpha (peg-IFNα) is approved for use in CHB patients although it is not the preferred therapy due to the occurrence of side effects. Furthermore, it is counter indicated for some patients such as those with liver cirrhosis (Saracco et al., 1994).

Untreated or off-treatment chronic patients are at risk to develop life threatening conditions such as fibrosis, cirrhosis, liver failure, and hepatocellular carcinoma (HCC). In 2015, 887,000 people died from HBV-related cirrhosis and liver cancer alone (WHO, 2017). The ultimate therapeutic goal in CHB is preventing these life-limiting outcomes and to achieve a functional cure characterized by the loss of surface antigen (HBsAg) and HBV-DNA in the blood off-treatment.

Hepatitis B virus functional cure will be achieved when the high viral load, the antigen burden and inadequate host immune responses are overcome and thus may need a broader therapeutic approach involving multiple targets, both viral and host. With regard to the latter, in-depth knowledge of the HBV life cycle is indispensable for identifying mechanisms, that are targetable with new therapeutics.

Part of the therapeutic approach may be to target the interface between viral proteins and cellular targets. The HBV viral proteins have pluripotent functions and our understanding of how they interact with host proteins is continuously evolving. The interactions of these viral factors with the host cell proteome are complex and helps to shape the cellular environment for the virus to replicate. In addition, cccDNA, the template of all viral mRNAs, behaves as a minichromosome and attracts a multitude of protein partners. However, all these reported interactions are scattered in literature, and currently there is no overview bringing together the interactome of HBV. This review aims to provide such an overview, from entry to viral release, it summarizes the known interactions between viral proteins and host proteins. Because cccDNA, HBc, and HBx have been described in many interactions, we focused the construction of an interactome network around these three entities.

## Interactions During the Early Phases of HBV Infection

The HBV particle consists of an incomplete 3.2 kb double-stranded (ds)DNA genome [relaxed circular DNA (RC-DNA)] packaged together with the viral polymerase in an icosahedral capsid assembled by HBV core (HBc) proteins (Summers et al., 1975). This nucleocapsid is enveloped by a lipid membrane studded with three forms of HBV surface antigen protein (collectively referred to as HBsAg) to compose the virus or Dane particle [reviewed by Bruss (2004)].

**FIGURE 1:**
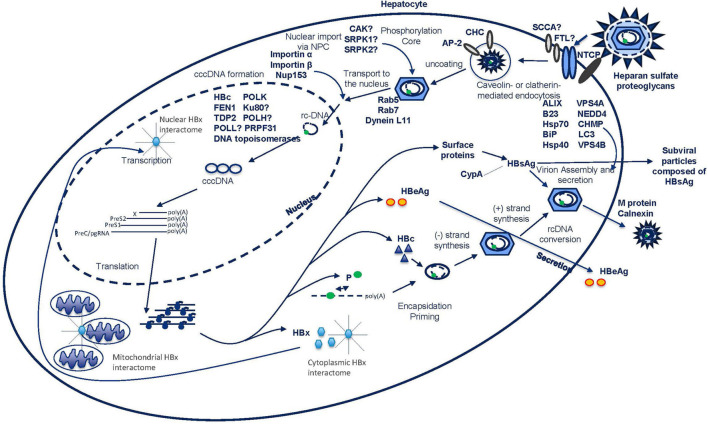
HBV life cycle from viral entry to release.

The life cycle of HBV begins upon its interaction with heparan sulfate proteoglycans (HSPGs) and subsequent binding to the sodium taurocholate co-transporting polypeptide (NTCP) receptor on the surface of the hepatocyte (Watashi et al., 2014; Yan et al., 2014; Figure 1). The interaction between virus and cell induces conformational changes of the membrane embedded myristoylated N-terminal preS1-domain of the viral large surface protein (L-HBsAg) leading to exposure of the receptor binding site for the NTCP receptor, which enables binding of the virus and entrance into the cell (Schulze et al., 2007, 2010; Yan et al., 2012, 2013, 2014; Nkongolo et al., 2014; Watashi et al., 2014). Recently, a crucial role in mediating HBV-NTCP internalization of epidermal growth factor receptor (EGFR) was published (Iwamoto et al., 2019). Besides the NTCP receptor, squamous cell carcinoma antigen 1 (SCCA1) and ferritin light chain (FTL) have also been identified as HBV co-receptors (Figure 1). Triple complexes of preS1, FTL, and SCCA1 were observed and overexpression assays with these proteins showed increased infection rates both *in vitro* and *in vivo* (Hao et al., 2012). The prevention of entry has been of interest as an antiviral target to circumvent viral spread by blocking *de novo* infection. In recent years molecules such as Myrcludex B (also known as bulevirtide), ezetimibe, cyclosporin derivates (CsA), and monoclonal antibodies against HBsAg epitopes were identified to interfere with this process (Gripon et al., 2005; Lucifora et al., 2013; Shimura et al., 2017).

The virus enters the cell by inducing endocytosis via caveolin-mediated endocytosis or via clathrin-mediated pathways (Macovei et al., 2010; Umetsu et al., 2018; Figure 1). In differentiated HepaRG cells, HBV infection has shown to be dependent on caveolin-mediated endocytosis. However, in Umetsu et al. (2018), the formation of a complex between the L-HBsAg, the clathrin heavy chain (CHC) and the clathrin adaptor protein-2 (AP-2) was described, suggesting an alternative endocytosis pathway (Figure 1). Indeed, inhibition of the clathrin-mediated pathway by silibinin and chlorpromazine has been reported to impair HBV uptake (Huang et al., 2012). Further work will be needed to understand the relative importance of these two pathways. After endocytosis, subsequent movement of the virus through the endocytic pathway is regulated by Rab proteins. These are guanosine triphosphatases (GTPases) that occupy specific endocytic compartments and direct endocytic vesicles to different cellular compartments. Silencing of Rab5 or Rab7, in contrast with Rab9 and Rab11, resulted in the inhibition of the early stages of HBV infection implying that the transport of virus to late endosomes is important for a successful infection (Macovei et al., 2013; Figure 1).

The precise location and timing of nucleocapsid release from the envelope remains unclear, but this process is required prior to nuclear entry. Transport of the nucleocapsid to the nucleus is facilitated by the microtubule network and the dynein L11 motor proteins through a direct interaction with the capsid (Osseman et al., 2018; Figure 1). In the nucleocapsid “uncoating” process, phosphorylation of the C-terminus of HBc destabilizes the capsid and allows the binding of importins α and β (Kann et al., 1999; Barrasa et al., 2001; Nguyen et al., 2008). Although a direct interaction has not been established, a number of kinases including core associated kinase (CAK), SR protein-specific kinase 1 (SRPK1) and SR protein-specific kinase 2 (SRPK2), have been reported to be involved in this phosphorylation process (Kau and Ting, 1998; Daub et al., 2002; Figure 1).

Once the nucleocapsid arrives at the nuclear pore complex (NPC), it can pass the complex as an intact particle (Pante and Kann, 2002; Fay and Pante, 2015). Interestingly, HBV seems to utilize a unique way of triaging immature from mature capsids at the level of the NPC as only mature capsids disassemble. In this process, importin β and Nup153 play a role via direct interaction with the capsid (Schmitz et al., 2010; Figure 1). Once through the NPC, the capsid is deposited in the nuclear basket where only mature capsids can pass. In the nucleus, the final uncoating, where capsid structures and viral DNA separate, takes place in an importin α and β-dependent manner (Gallucci and Kann, 2017).

## The cccDNA Minichromosome

Once inside the nucleus, the RC-DNA is converted into cccDNA (Summers et al., 1975; Tuttleman et al., 1986; Wu et al., 1990; Lieberman, 2016). Early research using duck hepatitis B virus (DHBV) showed that the cccDNA was in fact organized as a minichromosome similar to host chromatin and SV40 (Newbold et al., 1995). Further DHBV studies showed that *in vitro* between 1 and 56 copies cccDNA reside in the nuclei of infected cells (Kock et al., 2010). These copy numbers were slightly lower (1–17 copies/cell) in *in vivo* studies in ducks. Further it was determined that the half-life of DHBV cccDNA is between 35 and 57 days (Addison et al., 2002; Zhang et al., 2003) although shorter half-lives have described (Tuttleman et al., 1986; Wu et al., 1990; Newbold et al., 1995). *In vitro* kinetic studies were also done using HBV, cccDNA formation is an early life cycle event (Tuttleman et al., 1986) and it was shown that the cccDNA pool grows over the course of 3 days after which a stable pool is reached (5–12 copies/cell) with a half-life of about 40 days (Ko et al., 2014). Similar findings were done using woodchuck HBV (Dandri et al., 2000). Patient samples of HBV infected individuals showed that cccDNA copy numbers were much lower *in vivo* ranging from 0.01 to 9 copies/cell but at the same time had a much longer half-life of months to a year (Werle-Lapostolle et al., 2004; Bourne et al., 2007; Boyd et al., 2016; Huang et al., 2021). Interestingly, the size and half-life of the cccDNA pool in patients has been suggested to depend on the antigen status (Lythgoe et al., 2021) as much more cccDNA has been shown in HBeAg positive patients while only 0.002 copies/cell were observed in patients that showed HBsAg seroclearance (Werle-Lapostolle et al., 2004).

The cccDNA genome is transcribed to different viral RNAs coding for HBx (0.7-kb RNA), three forms of HBsAg (2.4-kb RNA encoding the large and 2.1-kb RNA encoding the middle and small HBsAg), pre-core protein or HBeAg (3.5-kb RNA) and the core and polymerase protein (pre-genomic RNA or pgRNA, 3.5-kb). This pgRNA also becomes incorporated in the nucleocapsid thereby providing the template for the viral polymerase to produce RC-DNA.

### Host Factors Involved in cccDNA Formation

Little is known about the host factors involved in the formation of the cccDNA. The L-HBsAg is not directly involved in cccDNA formation, but is part of a negative feedback mechanism in which high levels of surface protein shut down nuclear shuttling of mature nucleocapsids and direct the cell to produce virions instead (Summers et al., 1990). HBc is suggested to be present during the cccDNA formation process (Kock et al., 2010; Schreiner and Nassal, 2017) which is further evidenced by the fact that capsid modifiers inhibit cccDNA formation (Berke et al., 2017).

Several host factors have been reported to interact with HBV cccDNA during its formation and have quite diverse roles. The Flap endonuclease 1 (FEN1), an endonuclease that plays a role in DNA replication and repair, was shown to interact with RC-DNA in the nucleus and additionally could promote cccDNA formation *in vitro* (Kitamura et al., 2018; Figure 1). The discovery of a protein partner involved in DNA damage repair is coherent with the previous finding that this machinery is exploited by viruses to their own benefit (Schreiner and Nassal, 2017). Ku80, a component of non-homologous end joining DNA repair pathway, was essential for synthesis of cccDNA from dsDNA, but not from RC-DNA (Guo et al., 2012; Figure 1). In these processes, HBx could be an adaptor to link cccDNA formation with DNA damage response pathways, under the assumption that HBx is already present in the cell when cccDNA is being formed (Hodgson et al., 2012; Guo et al., 2014; Murphy et al., 2016; Niu C. et al., 2017). The link with the host DNA damage and repair machinery does not end with this interaction, the tyrosyl-DNA-phosphodiesterase (TDP2) also plays a partial role in cccDNA formation by releasing the viral transcriptase from the RC-DNA (Koniger et al., 2014; Cui et al., 2015; Figure 1). The host DNA polymerases K (POLK), H (POLH), and L (POLL) have all been reported to have a positive impact on cccDNA formation, however, the exact mechanism(s) is (are) not yet clear (Qi et al., 2016; Figure 1). In addition to DNA polymerases, knockout experiments showed the importance of cellular DNA ligase 1 and 2 in cccDNA formation (Long et al., 2017). Recently, it was shown that the plus-strand and the minus-strand require different cellular proteins. The plus-strand repair required proliferating cell nuclear antigen (PCNA), replication factor C (RFC) complex, DNA polymerase delta (POLδ), flap endonuclease 1 (FEN1), and DNA ligase 1 (LIG1) while the repair of the minus-strand only required FEN1 and LIG1 (Wei and Ploss, 2020). Also cellular DNA topoisomerases are required for cccDNA formation and amplification (Sheraz et al., 2019). Finally, pre-mRNA processing factor 31 (PRPF31) was identified as a cccDNA-associating factor involved in cccDNA formation (Kinoshita et al., 2017; Figure 1).

### The Interactome of the cccDNA

Similar to a cellular chromosome, the cccDNA is bound to histones to form a minichromosome. These host-derived histones (H2A, H2B, H3, and H4) provide, together with the viral HBc, the stable scaffold for the cccDNA to be supercoiled (Newbold et al., 1995; Chong et al., 2017). That being said, the role of HBc in both cccDNA formation and maintenance is still under investigation. For example, despite their involvement in several processes regarding cccDNA formation, maintenance and transcription, capsid modifying compounds do not eliminate the cccDNA pool (Berke et al., 2017) nor is HBc essential for transcription (Zhang et al., 2014).

On the cccDNA of Duck hepatitis B virus (DHBV), nucleosomes are non-randomly positioned, suggesting that, like host cellular chromatin, positioning of the nucleosomes and histone modifications of the cccDNA may regulate cccDNA transcription (Bock et al., 1994; Pollicino et al., 2006). Methylation, acetylation, phosphorylation or other posttranslational modifications (PTMs) of these cccDNA-bound histone tails can fine tune the gene expression by altering the chromatin structure (Tropberger et al., 2015). This change in structure can wind the chromatin more tightly to prevent access of transcription factors and repress gene transcription. On the other hand, histone modifications can also result in increased DNA accessibility, transcription factor binding and therefore promoting gene activation (Li et al., 2007; Voss and Hager, 2014). In addition, the minichromosome attracts several other partners, many of which are transcription factors that further determine whether the cccDNA is transcriptionally active or inactive (Table 1).

**TABLE 1:** List of known protein-cccDNA interactions associated with increased or decreased transcriptional regulation.

cccDNA minichromosome partner	Process	References
**Associated with Enhanced Replication – Verified Interactions**		
HBx	Required for replication and transcription.	Belloni et al., 2009
HBc	HBc binds to the CpG islands of HBV cccDNA.	Guo et al., 2011
CBP	HBx interacts and cooperates with CBP to modify chromatin dynamics and enhances CREB activity.	Pollicino et al., 2006; Belloni et al., 2009
P300	HBx increases amount of P300 recruited to promotors.	Belloni et al., 2009
PCAF	Recruited to the cccDNA after HBx binding to the minichromosome.	Belloni et al., 2009
LSD1/KDM1A	Recruited in an HBx-dependent manner, induces HBV replication and HBV transcription involves the demethylation of histone 3 lysine 9 (H3K9).	Alarcon et al., 2016
CREB/CREB1	Essential for HBV replication. It binds to the cAMP response elements (CREs) located at the X and preS2 promoters. Interaction with cccDNA dependent on CRTC1.	Tacke et al., 2005; Kim B.K. et al., 2008; Tang et al., 2014)
STAT1	Binds to cccDNA, binding impaired upon IFN treatment.	Belloni et al., 2012
STAT2	Binds to cccDNA, binding impaired upon IFN treatment.	Belloni et al., 2012
STAT3	May bind to enhancer I (ENI) and increase function.	Quarleri, 2014
Set1A/SETD1A	Recruited via a HBx-dependent manner, stimulates an active cccDNA epigenetic state by methylating histone 3 lysine 4 (H3K4) in viral HBV promoters.	Alarcon et al., 2016
CRTC1	Recruited to the preS2/S promotor for the activation of replication. Interaction with cccDNA dependent on CREB/CREB1.	Tang et al., 2014
KLF15	Activates S and HBc promotors and enhances replication when overexpressed.	Zhou et al., 2011
SIRT1	SIRT1 interacts with HBx and promotes the recruitment of HBx and other transcriptional factors to the cccDNA (specifically to the precore promoter), promoting the activation of HBV transcription (Deng et al., 2017). However, after IFNα treatment, SIRT1 is recruited to the cccDNA to repress transcription.	Belloni et al., 2012
RFX1	Binds the enhancer region upon doxorubicin treatment to promote replication.	Wang et al., 2018
RXRα	RXRα recruitment to the cccDNA in parallel with P300 recruitment	Zhang Y. et al., 2017
SP1	Several binding sites, depending on the site, the activity of SP1 is enhancing or inhibitory.	Quasdorff and Protzer, 2010
TBP	Binds the TATA box.	Quasdorff and Protzer, 2010
NRF1	Binds to the HBx promotor and positively regulates HBx transcription	Quasdorff and Protzer, 2010
C/EBP	Binds enhancer II (EnhII) and the HBc promotor. Low concentrations have a positive effect on replication while high concentrations evoke inhibition. Potentially also a repressor role.	Pei and Shih, 1990; Quasdorff and Protzer, 2010
PPAR	Increases transcription from several promotors.	Quasdorff and Protzer, 2010
FXR/NR1H4	Can bind EnhII and HBc regions to have a stimulating effect on transcription.	Quasdorff and Protzer, 2010
AP1	Binding to HBc promotor and shown to work in synergy with SIRT and HBx.	Quasdorff and Protzer, 2010; Ren et al., 2014
HNF1/HNF1A	Binding sites on the preS promotor. HNF1/HNF1A synergistically works with Oct1 and LRH-1/NR5A2 to enhance replication.	Zhou and Yen, 1991; Cai et al., 2003
LRH-1 (NR5A2)/hB1F	Transactivator of the EnhII and HBc regions. Synergy with HNF1/HNF1A.	Cai et al., 2003; Quasdorff and Protzer, 2010
HNF3	Several binding sites identified, binding seems to be associated with a stimulating effect.	Cai et al., 2003; Quasdorff and Protzer, 2010
HNF4/HNF4A	Stimulation of transcription from several promotors.	Cai et al., 2003; Quasdorff and Protzer, 2010
HLF	Stimulatory effect on the HBc regulatory region.	Ishida et al., 2000
FTF	Stimulatory effect on EnhII.	Ishida et al., 2000
Parvulin 14	Recruited to cccDNA in the presence of HBx to promote transcriptional activation.	Saeed et al., 2018
Parvulin 17	Recruited to cccDNA in the presence of HBx to promote transcriptional activation.	Saeed et al., 2018
Activation-induced cytidine deaminase (AID)	Interaction enhances cccDNA transcription.	Qiao et al., 2016
P19	Interaction enhances cccDNA transcription.	Qiao et al., 2016
**Associated with enhanced Replication – Potential Interactions**		
CRTC2	Enhances HBV transcription and replication by inducing PGC1α expression.	Tian et al., 2014
PGC1α	Induction of HBV transcription, potentially via FOXO1.	Quasdorff and Protzer, 2010; Tian et al., 2014
NF1	Three binding sites on HBV genome.	Ori et al., 1994; Quasdorff and Protzer, 2010
Oct1	Oct-1 and HNF-1 sites are necessary for liver-specific transcription of the preS1 promoter.	Zhou and Yen, 1991
EFC	Binding site identified in central HBc promotor.	Quarleri, 2014
**Associated with supressed Replication – Verified Interactions**		
HDAC1	Correlated with decline in replication.	Belloni et al., 2009; Levrero et al., 2009
	Actively recruited to the cccDNA under IFNα-treatment to repress transcription.	Belloni et al., 2012
YY1	Part of the transcriptional repressor complex PRC2. Actively recruited to the cccDNA under IFNα treatment to repress transcription.	Belloni et al., 2012
SETDB1	Repressing histone deacetylase.	Riviere et al., 2015; Alarcon et al., 2016
EZH2	Repression of cccDNA.	Salerno et al., 2020
HP1/CBX1	HP1/CBX1 proteins are recruited to the cccDNA through interaction with H3K9me3 and contribute to transcriptional repression.	Riviere et al., 2015
Spindlin 1/SPIN1	Inhibition of transcription from the cccDNA via epigenetic modulation.	Ducroux et al., 2014
APOBEC3G	May contribute to cccDNA editing. Antiviral effect through DNA and RNA packaging.	Nguyen et al., 2007; Luo et al., 2016
SP1	Several binding sites, depending on the site the activity of SP1 is enhancing or inhibitory.	Quasdorff and Protzer, 2010
TR4	Repressing function by inhibition of HNF4A mediated transactivation. Binds the HBc promotor.	Quasdorff and Protzer, 2010
HNF1/HNF1A	Binding site identified on EnhII. Binding associated with a decline in replication by induction of NF-κB/NFKB1.	Cai et al., 2003; Dai X. et al., 2014; Lin et al., 2017
HNF6	Inhibits gene expression and replication.	Hao et al., 2015
COUP-TF/NH2F1	Overexpression of COUP-TF/NH2F1 led to a decrease in replication via binding on NRRE in the enhancer and HBc regions.	Yu and Mertz, 2003
PRMT5	PRMT5-mediated histone H4 dimethyl Arg3 (H4R3me2) repressed cccDNA transcription. PRMT5-H4R3me2 interacted with HBc and the Brg1-based hSWI/SNF chromatin remodeler, which accounted for the reduced binding of RNA polymerase II to cccDNA.	Zhang W. et al., 2017
E4BP4/NFIL3	Associated with suppression of EnhII.	Ishida et al., 2000
NREBP	Inhibits core promotor activity by binding the NRE. Binding is inhibited by HBx.	Lee et al., 2019
ZHX2	Restriction factor that regulates HBV promoter activities and cccDNA modifications.	Xu et al., 2018
**Associated with supressed Replication – Potential Interactions**		
Prox1	Interacts with LRH-1/NR5A2 and downregulates LRH-1/NR5A2 mediated activation.	Quasdorff and Protzer, 2010
APOBEC3A	Upregulation by IFNα and lymphotoxin-β receptor resulted in cytidine deamination,	Lucifora et al., 2014
	apurinic/apyrimidinic site formation and finally cccDNA degradation.	
APOBEC3B	Upregulation by IFNα and lymphotoxin-β receptor resulted in cytidine deamination,	Lucifora et al., 2014
	apurinic/apyrimidinic site formation and finally cccDNA degradation.	
SIRT 3	Mediates cccDNA transcription. Repression lifted by HBx.	Ren et al., 2018

*Verified interactions are those protein-protein interactions that were identified using proteomics methods such as pull downs or yeast-2-hybrid. Potential interactions are those which have been shown using methods that strongly suggest an interaction (e.g., co-localization) but were not verified using pull-down methods.*

As previously mentioned, HBx and HBc proteins are bound to cccDNA. HBc has been described to modulate transcription from the cccDNA. Zlotnick et al. showed that the presence of HBc on a CpG island in the cccDNA can be linked to increased cccDNA activity, while methylation of the CpG island correlated with decreased cccDNA activity (Zlotnick et al., 2015). In addition, the presence of HBc appears to have a role in the maintenance of the structure of the cccDNA (Bock et al., 2001). Together these data suggest that HBc contributes to the epigenetic regulation of the cccDNA, which in turn contributes to its longevity.

### Modalities Acting on cccDNA

A role in viral rebound made cccDNA a target for new antiviral drug development. Success of such tactics relies on complete inhibition of cccDNA throughout the lifespan of the hepatocyte. A first approach is to target the formation of cccDNA, although it can be questioned how much benefit CHB patients will have of such a therapy in the event the cccDNA does not become reduced. Several molecules reported to act through this mechanism have been described in literature. However, to date, these molecules have either been stopped at pre-clinical stage or did not progress far in clinical trials (Cai et al., 2012; Liu et al., 2016). The only assets which encompass this capacity and are still under clinical investigation are the entry inhibitor bulevirtide and capsid assembly modulators. The latter are small molecules that accelerate capsid formation but turned out to have a dual mode of action in preventing cccDNA formation when added *in vitro* at early stages of infection (Berke et al., 2017; Vandenbossche et al., 2019). Secondly, a number of molecules have been described that silence the cccDNA, either by inhibiting cccDNA transcription [e.g., Tamibarotene (Nkongolo et al., 2019)] or by diminishing HBV RNA levels post-transcription (e.g., RNA destabilizers such as RG7834 (Mueller et al., 2019); RNA interference). Tamibarotene never made it to clinical trials for HBV, while RG7834 was stopped in Phase I. Transcriptional control of cccDNA expression may also be achieved by interfering with the function of HBx, HBc or an interaction partner. An example is the interference between HBx and DNA damage-binding protein 1 (DDB1). HBx was found to hijack DDB1 which in turn recruits the ubiquitylation machinery to send Structural Maintenance of Chromosomes protein 5/6(SMC5/6), a transcriptional repressor of cccDNA, to the proteasome for degradation. Two molecules, pevonedistat, a NEDD8-activating enzyme inhibitor, and nitazoxanide, a thiazolide anti-infective agent, have been shown to restore SMC5/6 levels and suppress viral transcription (Decorsiere et al., 2016; Sekiba et al., 2019a,b). Recently, epigenetic modifiers that specifically target viral factors involved in the regulation of cccDNA expression have been described and are currently being evaluated. Several selective inhibitors (e.g., C646) for histone acetyltransferase like CBP and P300 have been used to study the inhibitory effect on HBV transcription (Tropberger et al., 2015). The prodrug GS-5801 has also been shown to inhibit transcription from cccDNA by blocking the activity of lysine demethylase 5 (KDM5) (Gilmore et al., 2017). Although these observations show that silencing of HBV transcription is possible, the main throwback of most of these targets is the lack of desired selectivity for cccDNA and their potential to impact cellular processes.

**FIGURE 2:**
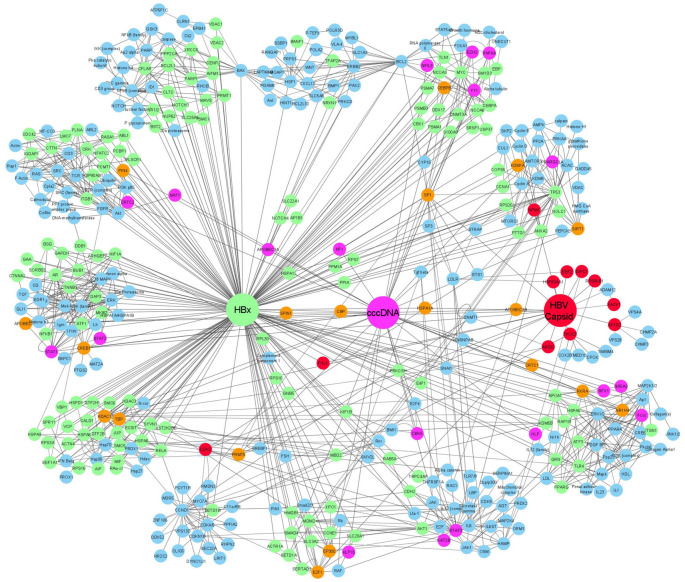
Gene association network showing the relationship between HBx, HBc, and HBV cccDNA interacting proteins. In the network, proteins which only interact with HBx are indicated in green, proteins which only interact with cccDNA are shown in pink, and proteins that only interact with HBc are shown in red. Proteins that were shown to interact with more than two of the founding nodes (cccDNA, HBc, and HBx) are depicted in orange. proteins that were extrapolated to connect to one or more interacting proteins are shown in blue.

Complete elimination of cccDNA by compromising the stability or the half-life of the molecule is often dubbed the “Holy Grail” of HBV research. Many molecules have been described that phenotypically reduce the quantity or transcription level of cccDNA. Recently, a small molecule, ccc_R08, with an unknown mode of action was shown to decrease the pool of cccDNA together with a decrease in viral transcripts and viral antigens in primary human hepatocytes (PHH) and in an HBV minicircle mouse model (Wang et al., 2019). In most instances, information on the exact mechanism of such molecules is lacking implying a need to conduct target deconvolution studies to identify the respective interaction partner or process. We created a cccDNA network map, not only to visualize the currently known cccDNA interacting proteins but also to be put alongside such exercises (Figure 2).

## The HBx Interactome

The interactome of HBx extends beyond its interaction with the cccDNA and associated proteins. Besides nuclear interaction partners, HBx also interacts with various proteins in the cytoplasm, the endoplasmic reticulum (ER) and the mitochondria (Henkler et al., 2001; Huh and Siddiqui, 2002; Belloni et al., 2009; Li et al., 2017; Figure 1). This may explain why this small viral protein (17-kDa) is not only involved in HBV replication, but is also shown to contribute to the development of HCC and interfere with cell cycle regulation, glucose metabolism, oxidative stress, calcium signaling, apoptosis and DNA repair (Luber et al., 1993; Waris et al., 2001; Bouchard et al., 2006; Benhenda et al., 2009; Table 2). The pivotal nature of HBx is demonstrated by Table 2 in which more than 250 HBx interaction partners are summarized. However, it does need to be mentioned that some of these interactions may be very weak or very brief and their relevance may be limited.

**TABLE 2:** HBx interacting proteins listed together with the cellular processes or pathways in which they are involved.

Interaction partner	Cellular Process	References
RPB5	Transcriptional machinery	Cheong et al., 1995
TFIIB	Transcriptional machinery	Lin et al., 1997
TBP	Transcriptional machinery	Qadri et al., 1995
TFIIH	Transcriptional machinery	Qadri et al., 2011
CBP	Coactivator	Cougot et al., 2007
P300	Coactivator	Cougot et al., 2007
PCAF	Coactivator	Chan et al., 2013
ATF/CREB	Transcription factor	Maguire et al., 1991
ATF3	Transcription factor	Barnabas and Andrisani, 2000
ICERIIgamma	Transcription factor	Barnabas et al., 1997; Barnabas and Andrisani, 2000
gadd153/Chop10	Transcription factor	Barnabas et al., 1997
c/EBPA	Transcription factor	Choi et al., 1999
NF-IL-6	Transcription factor	Barnabas et al., 1997
ETS/ERG^#^	Transcription factor	Qin et al., 2013
EGR/EGRF1	Transcription factor	Yoo and Lee, 2004
SMAD4	Transcription factor	Shi et al., 2016
Oct1	Transcription factor	Antunovic et al., 1993
RXR	Transcription factor	Kong et al., 2000
P53	Transcription factor. Induces destabilization of HBx.	Belloni et al., 2009; Xian et al., 2010; Iyer and Groopman, 2011
PRMT1	Relieves PRMT1 suppression from viral replication.	Benhenda et al., 2013
Spindlin1	Interaction with HBx relieves repression by Spindlin1. Knockdown induced an increase in HBV transcription and H3H4 trimethylation on the cccDNA.	Ducroux et al., 2014
PP1	HBx interferes with the inactivation of CREB/CREB1 by PP1.	Cougot et al., 2012
JMJD5	Interaction with HBx facilitates HBV replication through the hydroxylase activity of JMJD5.	Kouwaki et al., 2016
DDB1	Recruited resulting in SMC5/6 degradation.	Decorsiere et al., 2016
SMC5/6	Recruited to the ubiquitin machinery to be degraded to enhance transcription.	Decorsiere et al., 2016
hepatocystin	May be an antiviral pathway, hepatocystin seems to accelerate HBx degradation.	Shin et al., 2013
Clathrin heavy chain	Unknown	Shin et al., 2013
HSPA5	Unknown	Shin et al., 2013
HSPA9	Unknown	Shin et al., 2013
CALD1	Unknown	Shin et al., 2013
HSPA8	Unknown	Shin et al., 2013
XRCC6	Unknown	Shin et al., 2013
PDIA4	Unknown	Shin et al., 2013
PRKCSH	Unknown	Shin et al., 2013
HSPA6	Unknown	Shin et al., 2013
DDX17	Unknown	Shin et al., 2013
HSPA1L	Unknown	Shin et al., 2013
HSPA1A	Unknown	Shin et al., 2013
SIRT1	SIRT1 interacts with HBx thereby enabling HBx-induced transcriptional activity cccDNA.	Srisuttee et al., 2012; Deng et al., 2017
Set1A/SETD1A^#^	Recruited by HBx to cccDNA to increase transcription.	Alarcon et al., 2016
LSD1^#^	Bound to viral promotors.	Alarcon et al., 2016
CRTC1	Interaction associated with increased transcription.	Tang et al., 2014
CPAP/CENPJ	Promotes HBx-mediated cell proliferation and migration in a SUMO-dependent manner.	Yang et al., 2013
CREB/CREB1	Upregulated via HBx-CREB/CREB1 interaction.	Yang et al., 2013
CRM1/XPO1	Potential activation of CRM1/XPO1 and role in HBx-mediated carcinogenesis.	Forgues et al., 2001
NFκB^#^	Relocalization via NES motif.	Forgues et al., 2001
VISA/MAVs	Disruption of VISA/MAVs and downstream interacting proteins thereby impairing IFN signaling.	Wang X. et al., 2010
MDA5	Impairment of IFN signaling.	Wang X. et al., 2010
GRP78	Role in HCC via suppression of eIF2α phosphorylation, inhibited expression of ATF4/CHOP/Bcl-2, and reduced cleavage of PARP.	Li et al., 2017
AKT1	Cell proliferation, abrogation of apoptosis and tumorigenic transformation of cells.	Khattar et al., 2012
Bcl-2	Management of calcium levels to benefit viral replication.	Geng et al., 2012
BCL2L1	Management of calcium levels to benefit viral replication.	Geng et al., 2012
HDAC1	Repression insulin-like growth factor binding protein-3. HBx also induces HDAC1.	Pollicino et al., 2006; Yoo et al., 2008; Shon et al., 2009
SP1^#^	HBx induces deacetylation of SP1.	Pollicino et al., 2006; Yoo et al., 2008; Shon et al., 2009
HIF1a	HBx aids the MTA1/HDAC complex in stabilizing HIF1a.	Yoo et al., 2008
USP-15	USP-15 mediated deubiquitylation protects HBx from proteasomal degradation.	Su et al., 2017
PARP1	DNA damage and repair, carcinogenesis.	Na et al., 2016
Cardiolipin (lipid)	Mitochondrial membrane permeabilization.	You et al., 2019
Prdx1	Peroxiredoxin interfaces with HBV-RNA to promote RNA decay, potentially HBx rescues this event.	Deng et al., 2018, 2019
Parvulin 14/PIN4	Interaction with HBx in nucleus, cytoplasm and mitochondria to enhance HBx stability, translocation to the nucleus and mitochondria to increase HBV replication.	Saeed et al., 2018
Parvulin 17	Interaction with HBx in nucleus, cytoplasm and mitochondria to enhance HBx stability, translocation to the nucleus and mitochondria to increase HBV replication.	Saeed et al., 2018
14-3-3ζ	Interaction found in HCC cells, involvement of AKT pathway.	Tang et al., 2018
c-myc	Oncogenesis	Lee et al., 2016
Orail protein	Calcium metabolism	Yao et al., 2018
HMGB1	Autophagy	Fu et al., 2018
FXR/NR1H4	Transactivation FXR/NR1H4, oncogenesis	Niu Y. et al., 2017
PP2Ac/PP2CA	Cell cycle and apoptosis	Gong et al., 2016
SMYD3	Involved in AP1 activation	Hayashi et al., 2016
P62	Glucose metabolism	Liu B. et al., 2015
TLR4	Tumorigenesis	Wang et al., 2015
BST-2^@^	HBV restriction factor	Lv et al., 2015
MBD2	Involved in epigenetics of histones, potentially in HCC.	Liu X.Y. et al., 2015
TRUSS/TRPC4AP	May be linked to pathological sequelae of HBV.	Jamal et al., 2015
MKI67	Cell proliferation	Zhang et al., 2015
ENPEP	Cell proliferation	Zhang et al., 2015
MIF	Cell proliferation	Zhang et al., 2015
PYY	Cell proliferation	Zhang et al., 2015
NOLC1	Cell proliferation	Zhang et al., 2015
CDC42	Cell adhesion	Zhang et al., 2015
IQGAP1	Cell adhesion	Zhang et al., 2015
LMO7	Cell adhesion	Zhang et al., 2015
ACTN4	Cell adhesion	Zhang et al., 2015
CTNNA2	Cell adhesion	Zhang et al., 2015
MYH2	Cell adhesion	Zhang et al., 2015
FILAMIN	Cell adhesion	Zhang et al., 2015
ITGB1	Cell adhesion	Zhang et al., 2015
TLN1	Cell adhesion	Zhang et al., 2015
NRXN1	Cell adhesion	Zhang et al., 2015
CDH2	Cell migration	Zhang et al., 2015
NOTCH4	Angiogenesis	Zhang et al., 2015
CTNNB1	Angiogenesis	Zhang et al., 2015
ANXA2	Angiogenesis	Zhang et al., 2015
ATP5B	Angiogenesis/cell adhesion	Zhang et al., 2015
PSMC4	Protein degradation	Zhang et al., 2015
PSMB3	Protein degradation	Zhang et al., 2015
VDAC1	Anion transport	Zhang et al., 2015
VDAC2	Anion transport	Zhang et al., 2015
SLC25A3	Transport	Zhang et al., 2015
S100A9	Viral reproduction	Zhang et al., 2015
SLC25A5	Viral reproduction	Zhang et al., 2015
SLC25A10	Metabolic process	Zhang et al., 2015
SLC20A1	Signal transduction	Zhang et al., 2015
SLC3A2	Immune system process	Zhang et al., 2015
RAP1B	Signal transduction	Zhang et al., 2015
RAB10	Signal transduction	Zhang et al., 2015
RAB11B	Signal transduction	Zhang et al., 2015
RAB5A	Signal transduction	Zhang et al., 2015
FIS1	Programmed cell death	Zhang et al., 2015
KIF1B	Programmed cell death	Zhang et al., 2015
DAP3	Induction of apoptosis	Zhang et al., 2015
VIM	Apoptosis	Zhang et al., 2015
JUP	Cell migration	Zhang et al., 2015
RPS7	Viral reproduction	Zhang et al., 2015
RPS10	Viral reproduction	Zhang et al., 2015
RPS16	Viral reproduction	Zhang et al., 2015
RPS20	Viral reproduction	Zhang et al., 2015
RPL30	Viral reproduction	Zhang et al., 2015
RPL38	Viral reproduction	Zhang et al., 2015
BANF1	Viral reproduction	Zhang et al., 2015
AP1B1	Viral reproduction	Zhang et al., 2015
BSG	Immune system process	Zhang et al., 2015
ACTR1A	Cell cycle	Zhang et al., 2015
SRSF1	mRNA processing	Zhang et al., 2015
DDB1	Wnt receptor signaling pathway	Zhang et al., 2015
ATP5C1	Oxidative phosphorylation	Zhang et al., 2015
PCMT1	Protein methylation	Zhang et al., 2015
PPIA	Viral reproduction	Zhang et al., 2015
HIST2H2BE	Nucleosome assembly	Zhang et al., 2015
PCBP1	Metabolic process	Zhang et al., 2015
GAPDH	Glycolysis	Zhang et al., 2015
HSP90AB1	Regulation of signaling pathway	Zhang et al., 2015
COXIII^@^	Mitochondrial function	Li et al., 2015; Zou et al., 2015
ECSIT	Involved in IL-1β induction of NF-κB activation.	Chen et al., 2015
Skp2	Cell cycle deregulation and transformation.	Kalra and Kumar, 2006
PSMA7/XAPC7	Proteasome	Huang et al., 1996
PSMC1	Proteasome	Zhang et al., 2000
PSMA1	Proteasome	Hu et al., 1999
PLSCR1	Unknown	Yuan et al., 2015
GRN	Unknown	Yuan et al., 2015
SPRY1	Unknown	Yuan et al., 2015
NKD2	Unknown	Yuan et al., 2015
SYVN1	Unknown	Yuan et al., 2015
NOTCH3	Unknown	Yuan et al., 2015
LAMC3	Unknown	Yuan et al., 2015
SERTAD1	Unknown	Yuan et al., 2015
GAA	Unknown	Yuan et al., 2015
USP37	Cell cycle progression	Saxena and Kumar, 2014
E4F1	P53-dependent growth arrest	Dai Y. et al., 2014
Pregnane X receptor	Potentially involved in carcinogenesis	Niu et al., 2013
apoA-I	HBV secretion	Zhang et al., 2013
hBubR1/BUB1	Genomic stability	Chae et al., 2013
c-FLIP_*L*_	Apoptosis	Kim and Seong, 2003
c-FLIP_*S*_	Apoptosis	Kim and Seong, 2003
AIF	Apoptosis	Liu et al., 2012
AMID	Apoptosis	Liu et al., 2012
AIB1	NFκB signaling	Hong et al., 2012
eEF1A1	Actin bundling	Lin et al., 2012
VCP	NFκB signaling	Jiao et al., 2011
RPS3a	NFκB signaling	Lim et al., 2011
Gli1	Hedgehog signaling	Kim et al., 2011
Phosphor-p65	NFκB signaling	Shukla et al., 2011
IPS-1	RIGI signaling	Kumar et al., 2011
C/EBPα	Insulin signaling	Kim K. et al., 2010
PTTG1^@^	Tumorigenesis	Molina-Jimenez et al., 2010
Cul1^@^	Tumorigenesis	Molina-Jimenez et al., 2010
TNFR1^@^	NFκB signaling	Kim J.Y. et al., 2010
Cortactin	Cytoskeletal	Feng et al., 2010
Yes1	Cell growth and survival, apoptosis, cell-cell adhesion, cytoskeleton remodeling, and differentiation.	Feng et al., 2010
CRK-D2	Regulates cell adhesion, spreading and migration.	Feng et al., 2010
c-Src	Signal transduction	Feng et al., 2010
Y124	Unknown	Feng et al., 2010
RasGAP	GTPase, unknown	Feng et al., 2010
Abl	Cell growth and survival, cytoskeleton remodeling in response to extracellular stimuli, cell motility and adhesion, receptor endocytosis, autophagy, DNA damage response and apoptosis.	Feng et al., 2010
ITSN-D1/ITSN1	Unknown	Feng et al., 2010
Abl2	Cell growth and survival, cytoskeleton remodeling in response to extracellular stimuli, cell motility and adhesion and receptor endocytosis.	Feng et al., 2010
OSF/OSTF1	Cell adhesion	Feng et al., 2010
Tec	Cytoskeletal, adaptive immunity	Feng et al., 2010
PIG2/GAMT	Carcinogenesis	Feng et al., 2010
ARH6	DNA damage	Feng et al., 2010
EFS	Cell adhesion	Feng et al., 2010
RHG4	Unknown	Feng et al., 2010
VINE-D1	Cytoskeletal	Feng et al., 2010
VINE-D3	Cytoskeletal	Feng et al., 2010
HSP72/ASPA1A	Chaperone	Wang et al., 2008
C/EBPbeta	Phase II detoxifying pathways.	Cho et al., 2009
DNMT3A	Epigenetic modifications	Zheng et al., 2009
Bax	Apoptosis	Kim H.J. et al., 2008
VBP1	NFκB signaling	Kim S.Y. et al., 2008
betaPIX	Rec1 signaling	Tan et al., 2008
HBXIP	Centrosome and spindle formation.	Wen et al., 2008
Pin1	Carcinogenesis	Pang et al., 2007
AR	Gene expression	Zheng et al., 2007
PP2Calpha	Carcinogenesis	Kim et al., 2006
cyclin E/A	Cell cycle regulation	Mukherji et al., 2007
vinexin-beta	Cytoskeletal organization	Tan et al., 2006
MIF	Apoptosis	Zhang et al., 2006
Jab1/cops5	AP1 signaling	Tanaka et al., 2006
GNbeta5	Unknown	Lwa and Chen, 2005
p120E4F	Mitosis and cell cycle	Rui et al., 2006
Hepsin	Apoptosis	Zhang et al., 2005
Hsp60	Apoptosis	Tanaka et al., 2004
PPARgamma	Apoptosis	Choi et al., 2004
ASC-2	Carcinogenesis	Kong et al., 2003
E2F1	Carcinogenesis	Choi et al., 2002
NF-AT1/NFATC2	Calcium metabolism	Carretero et al., 2002
Tbp1	Transcription	Barak et al., 2001
NF-IL6	IL6 signaling	Ohno et al., 1999
Jak1	JAK/STAT signaling	Lee and Yun, 1998
XAP-1/UVDDB/DDB1	DNA damage repair, carcinogenesis.	Becker et al., 1998
RPB5	Transcription	Lin et al., 1997
HVDAC3/HDAC3^@^	HBx colocalized with HVDAC3/HDAC3 at the mitochondria.	Rahmani et al., 2000
AP2α^#^	HBx modulates SPHK1 via AP2α.	Lu et al., 2015
SetDB1^#^	HBx relieves SETDB1-mediated H3K9me3 induced silencing of cccDNA.	Riviere et al., 2015
HP1/CBX1^#^	HBx relieves HP1/CBX1 induced silencing of cccDNA.	Riviere et al., 2015
Id-1^#^	Id-1 destabilizes HBx by facilitating the interaction between ubiquitinated HBx and the proteasome.	Ling et al., 2008
HDM2/MDM2^#^	Promotes NEDDylation of HBx thereby enhancing its stability.	Liu et al., 2017
WDR5	Facilitates recruitment of HBx to promotor regions.	Gao et al., 2020
CBFβ	Blocks HBx function in promoting replication.	Xu et al., 2019
inhibitors of differentiation 1 (Id1)	Interaction accelerates degradation of these proteins.	Xia et al., 2020
inhibitors of differentiation (Id3)	Interaction accelerates degradation of these proteins.	Xia et al., 2020
PRPF31	Potential enhancement of cccDNA transcription through this interaction.	Kinoshita et al., 2017

*^#^ Unknown if this pertains a real protein-protein interaction; @ potential interaction, evidenced by co-localization.*

Besides the transcriptional modulation of cccDNA, HBx has also been described to modulate gene expression of multiple proteins involved in signaling pathways such as the AKT serine/threonine kinase 1 (AKT1), Ras-Raf-mitogen-activated protein (MAP) kinase, MAPK8/pSMAD3L, (TβRI)/pSMAD3C, nuclear factor-kappa B (NF-kB) pathways and potential restriction factors such as STIM1, zinc finger E-box binding homeobox 2 (ZEB2), and proteasome activator subunit 4 (PSME4) (Benn et al., 1996; Klein and Schneider, 1997; Waris et al., 2001; Yoo et al., 2008; Zhang et al., 2012; Liu et al., 2014; Lu et al., 2015; Rawat and Bouchard, 2015; Wu et al., 2016; Yu et al., 2016; Cheng et al., 2018; Zheng et al., 2019; Minor et al., 2020; Table 2). Interestingly, HBx expression itself is also influenced by cellular proteins, for example, NRF1 has shown to bind the HBx promotor to activate it in contrast to ATF2, which showed the opposite effect (Choi et al., 1997; Tokusumi et al., 2004; Quasdorff and Protzer, 2010).

## The HBc Interactome

HBc is mostly known as the building block of the HBV capsid (Summers et al., 1975) but in recent years it has been shown that its function is not limited to this and also plays a role in cccDNA stability, transcription and epigenetic regulation (Newbold et al., 1995; Bock et al., 2001; Zlotnick et al., 2015; Chong et al., 2017), evasion of antiviral mechanisms (Lucifora et al., 2014), reverse transcription (Tan et al., 2015), cellular trafficking (Schmitz et al., 2010; Yang et al., 2014), genomic replication (Lott et al., 2000), and viral egress (Bardens et al., 2011). The field is also discovering more and more that HBc expression is extensively regulated by core promotor regulation, core mRNA modulation and post-translational modifications which highlights its importance in the life cycle (Buckwold et al., 1997; Sohn et al., 2006; Kohno et al., 2014; Qian et al., 2015; He et al., 2016; Bartusch et al., 2017; Lubyova et al., 2017; Heger-Stevic et al., 2018; Makokha et al., 2019).

Initially, the impact on the capsid made HBc an appealing drug-target (Berke et al., 2017). However, given that there is also an interplay with cccDNA and HBx these molecules may have more far-reaching consequences. As more protein interactions between HBc and the host are elucidated, we also compiled the interactome of the core protein and linked it to the HBx and cccDNA interactomes (Figure 2).

## A cccDNA and HBx Gene Association Network: Expanding the Potential cccDNA and HBx Interactome

Tables 1–3 summarize what is currently known in the literature (manual curation) about cccDNA, HBx protein, and HBc protein-DNA and protein-protein interactions, respectively. However, to utilize this information to predict and potentially identify new protein interactions, network pathway analysis was performed (Ingenuity Pathway Analysis, IPA, Qiagen). IPA enables gene network generation from the Ingenuity Knowledge Base, a data repository of biological interactions and functional annotations.

**TABLE 3:** HBc interacting proteins listed together with the cellular processes or pathways in which they are involved.

Protein interaction partner	Process	References
Filamin B	Interaction promotes replication.	Li et al., 2018
Nucleophosmin	Promotion of capsid assembly.	Jeong et al., 2014
APOBEC3B	Potential editing of DNA during reverse transcription.	Chen et al., 2018
SRSF10	Acts as a restriction factor that regulates HBV RNAs levels.	Chabrolles et al., 2020
p70 ribosomal S6 kinase S6K1	HBc modulates phosphorylation levels of S6K1.	Wang et al., 2021
PRMT5	Methylation of the cccDNA.	Zhang Y. et al., 2017
Importin β	Capsid assembly.	Chen et al., 2016
NIRF	Inhibition of infection.	Qian et al., 2015
Hsp90	Catalyzes the formation of the capsid by binding HBc dimers.	Shim et al., 2011
hypermethylated in cancer 2 HIC2	Unknown	Lin et al., 2006
eukaryotic translation elongation factor 2 EEF2	Unknown	Lin et al., 2006
acetyl-coenzyme A synthetase 3	Unknown	Lin et al., 2006
DNA polymerase gamma POLG	Unknown	Lin et al., 2006
putative translation initiation factor SUI	Unknown	Lin et al., 2006
chemokine C-C motif receptor 5	Unknown	Lin et al., 2006
mitochondrial ribosomal protein L41 MRPL41	Unknown	Lin et al., 2006
kyot binding protein genes	Unknown	Lin et al., 2006
RanBPM	Unknown	Lin et al., 2006
HBeAg-binding protein 3 HBEBP3	Unknown	Lin et al., 2006
programmed cell death 2 PDCD2	Unknown	Lin et al., 2006
SP1	Inhibition of anti-viral mechanism of Mitochondrial antiviral signaling protein MAVS.	Li et al., 2021
coactivator cAMP response element CRE	aspecific interaction enhances the binding of the cAMP response element-binding protein CREB to CRE.	Xiang et al., 2015
C12 protein	Unknown	Lu et al., 2005
SRPK2	Mediate HBV core protein phosphorylation, unknow role in viral infection.	Daub et al., 2002
SRPK1	Mediate HBV core protein phosphorylation, unknow role in viral infection.	Daub et al., 2002
NXF1	Involved in cellular trafficking of HBc	Yang et al., 2014
TREX transcription/export complex	Involved in cellular trafficking of HBc	Yang et al., 2014
BAF200C	Evasion of host anti-viral mechanisms.	Li et al., 2019
BAF200	Evasion of host anti-viral mechanisms.	Li et al., 2019
Hsp70	Promotes capsid formation.	Seo et al., 2018
MxA	Immobilizes HBc in perinuclear compartiment, possible interference with capsid formation.	Li et al., 2012
Hdj1	Accelerated degradation of the viral core and HBx proteins	Shon et al., 2009
hTid1	Accelerated degradation of the viral core and HBx proteins	Shon et al., 2009
Skeletal muscle and kidney enriched inositol phosphatase SKIP	Interaction induces HBV gene suppression.	Hung et al., 2009
Atg12	Modulation of authophagy.	Doring et al., 2018
Np95/ICBP90-like RING finger protein NIRF	Potentially involved in maturation of the virus.	Qian et al., 2012
PTPN3	Supression of HBV gene expression.	Hsu et al., 2007
APOBEC3G	APOBEC3G is potentially incorporated in the virion through this interaction.	Zhao et al., 2010
PTPN3	May be bound within the capsid, function unknown.	Genera et al., 2021
PML	Link between DNA damage response and HBV replication. HBc co-localizes in PML-NBs.	Chung and Tsai, 2009
HDAC1	Link between DNA damage response and HBV replication. HBc co-localizes in PML-NBs.	Chung and Tsai, 2009
GIPC1	Unknown.	Razanskas and Sasnauskas, 2010
Activation-induced cytidine deaminase AID	HBc is the link between AID and cccDNA.	Qiao et al., 2016
Gamma-2 adaptin	Endosomal function and viral egress.	Rost et al., 2006
Nedd4	Virus production.	Rost et al., 2006
ABP-276/278	Affects viral replication via unknown mechanism.	Huang et al., 2000
B23	Unknown	Ludgate et al., 2011
I2PP2A	Unknown	Ludgate et al., 2011
APOBEC3B	APOBEC3B A3B displays dual inhibitory effects on HBV core-associated DNA synthesis.	Zhang et al., 2008
Receptor of activated protein kinase C 1 RACK1	Interference in normal TNF-a-regulated apoptosis.	Jia et al., 2015
nucleophosmin B23	Unknown	Lee et al., 2009
CDK2	Role in disassembly of the nucleocapsid.	Liu et al., 2021
E2F1	HBc reduced the DNA-binding ability of E2F1 to the binding site of the p53 promoter and respectively represses expression of p53.	Kwon and Rho, 2003
SIRT7	cccDNA expression modulation, HBc functions as a bridge between cccDNA and SIRT7.	Yu et al., 2021
HBs	Interaction prevents nuclear translocation to HBc.	Zajakina et al., 2014

To generate gene association networks, the HBx, HBc, and HBV cccDNA interacting proteins were individually analyzed to create three separate network schemes. The database was filtered and core analysis performed to only query the following: (1) Species = Human, (2) Molecules per networks = 35, Networks per analysis = 10, (3) Node Types = All, (4) Data Source = All, (5) Confidence = Experimentally Observed, (6) Species = Human, (7) Tissues and Cell Types = Liver, Hepatocytes, Hepatoma Cell Lines not otherwise specified, HuH7 cell line, Hep3B cell line, HepG2 cell line and “Other” Hepatoma cell lines, and (8) Mutation = All.

If proteins selected as network “seeds” were not apparently connected or networks had less than 35 gene products, IPA added proteins from the IPA Knowledge data base to maximize the connectivity of the “seed” molecules within the filter limits. We also filtered out those proteins that were only interacting with either cccDNA, HBx, or HBc and had no extrapolated nodes. This kept the networks to a manageable size and reduced redundancy while deriving as much as possible biological context from the analysis. When adding molecules from the knowledge database, IPA uses a connectivity metric (edge-weighted spring layout) that prioritizes molecules that have the greatest overlap with the existing network. This means that the organization of the network in clusters is not based on proteins sharing similar pathways but is based on the number of described interactions in between those proteins. Upon completion of the IPA network generating algorithm, two networks were produced showing both “direct” and “indirect” relationships for either HBx, HBc, or HBV cccDNA interacting proteins. These were then merged and exported to Cytoscape 3.7.1 using an edge weighted spring layout to create the final network illustration showing the relationship between HBx, HBc, and HBV cccDNA interactingproteins (Figure 2).

In the network, proteins that interact with HBc are depicted in red, those interacting with cccDNA in pink and those interacting with HBx in green. We also highlighted those proteins that were described to interact with 2 or more of our founding (HBc, HBx, and cccDNA) nodes (Figure 2). Nineteen proteins are identified (P300, TBP, PIN4, CBP, SPIN1, CEBPB, SP1, CRTC1, RXRA, NR1H4, KDM1A, HSPA1A, APOBEC3G, APOBEC 3B, CREB1, PRMT5, HDAC1, E2F1, and SIRT1) as interacting proteins of HBx, HBc, and cccDNA. These are interesting because these components may be a driving force in cccDNA transcription and maintenance. Moreover, these may be interesting proteins for further functional research as they seem to play a connecting role in the viral life cycle (Figure 2). Most of these proteins, 12 out of 19, are regulators of transcription, for example, TBP and CRTC1 are both involved in transcription initiation; CREB1 and E2F1 are enhancers of transcription; P300, CBP, SPIN1, SP1, PRMT5, HDAC1, and SETD1B are all epigenetic modifiers that can influence the chromatin to a specific transcriptionally accessible, active state. Finally, PIN4 was described as a chromatin remodeler. Any of these proteins could be a potentially interesting target to influence transcriptional status of cccDNA. Notable is that all these transcription-related proteins have interactions with HBc, hereby confirming a role for HBc beyond capsid assembly. Also interesting is the occurrence of two APOBEC proteins as partners for HBx, HBc and cccDNA. APOBEC proteins play a role in anti-viral immunity (Stavrou and Ross, 2015) and is a means of the cell to counteract the effect of infection (Lucifora et al., 2014).

Through the IPA approach, the network was expanded from just those proteins with known interactions with HBx, HBc, or cccDNA to an additional 210 proteins (indicated in light blue) which may play a role in protein-protein or protein-cccDNA interactions. While the interactions itself are verified in literature, their involvement in the HBV pathology and viral replication cycle is not confirmed yet. Hence, these proteins provide an interesting starting point for further research. Analysis of the network shows that via these interacting proteins, HBV also taps into host pathways such as cell cycle, cell signaling, DNA repair, transcription regulation and apoptosis. This is not surprising as many of these processes have been described in relation to HBV already. However, this is the first time description of proteins which may be involved in these processes and how they relate to the cccDNA minichromosome, HBc or HBx. An additional interesting observation is that many heat shock proteins (HSP) (Hsp70, Hsp90, Hsp27, HSPD1, HSPA1A, HSPA1B, HSPA8, HSPA9, HSPA1L, HSP90AB1, HSPA5, and HSPA6) were observed as interacting proteins in this network. Literature has already described that viruses rely on host HSPs for viral protein folding and induce overexpression of HSPs in the infected cells (Bolhassani and Agi, 2019). Moreover, several HSPs were associated with some viral particles (Fust et al., 2005; Bolhassani and Agi, 2019). In HBV, downregulation of Hsp70 and Hsp90 by small interfering RNA significantly inhibited HBV production. Furthermore, also a significant reduction of HBV secretion could be observed in HepG2.2.15 cells treated with an Hsp90 inhibitor (Liu et al., 2009; Bolhassani and Agi, 2019). Further research will be required to confirm the additional protein partners identified in this network analysis.

## Interactions During the Late Phases of HBV Infection

In HBV infection, besides the budding of virions, there is also the shedding of an excess amount of subviral particles (Figure 1). These particles are non-infectious 22 nm spheres or filaments of variable length consisting solely of the HBsAg envelope protein, which may be expressed from either cccDNA or HBV DNA that is integrated into the human genome (Heermann et al., 1984; Figure 1). Budding of infectious virus and shedding of subviral particles happen via distinct pathways (Selzer and Zlotnick, 2015).

Although redundant in viral assembly, the M-protein and its interaction with calnexin has been shown to be involved in the secretion of subviral particles (Werr and Prange, 1998). In the cytoplasm, HBsAg interacts with cyclophilin A (CypA) and stimulates the extracellular secretion of CypA (Tian et al., 2010; Figure 1). Interestingly, it seems that the presence of CypA reciprocally stimulates HBsAg secretion, as inhibitors against CypA reduce the amount of secreted HBsAg (Phillips et al., 2015).

To construct new virions, the pgRNA is packaged together with the viral polymerase in the nucleocapsid, which is formed in the cytoplasm by assembly of 120 HBc dimers (Lambert et al., 2007). Although not well understood, the interaction between HBc dimers and cellular protein nucleophosmin (B23) was shown to promote this assembly (Jeong et al., 2014; Figure 1). This nucleocapsid is surrounded by a cellular lipid layer embedded with three viral S glycoproteins, which originate from the endoplasmic reticulum (Bruss, 2007). Virion assembly depends solely on the L-protein, whereas the S-protein is required but not sufficient, and the M-protein is redundant (Bruss, 2004). To aid in building this unusual composition, Hsp70, and mammalian BiP were described as interaction partners of the L-protein *in vitro* and *in vivo* (Loffler-Mary et al., 1997; Lambert and Prange, 2003; Wang Y.P. et al., 2010; Figure 1). In the assembly of the mature virion, the S-protein needs to interact with the nucleocapsid (Loffler-Mary et al., 2000).

Once the mature virion is formed, it is ready to bud on the surface of the cells. The whole orchestration of this process is not clear at all, let alone accurately described in terms of interacting proteins. HBV makes use of the ESCRT, a machinery essential for the sorting of cellular cargo proteins in multivesicular bodies (Bardens et al., 2011). In this process, aryl hydrocarbon receptor interacting protein (AIP1)/ALIX and vacuolar protein sorting 4 homolog B (VPS4B) were found to colocalize with HBV particles (Kian Chua et al., 2006; Watanabe et al., 2007; Figure 1). Also, expression of dominant negative mutants of ESCRT-III complex-forming charged multivesicular body protein (CHMP) proteins (CHMP3, 4B, and 4C), as well as vacuolar protein sorting 4 homolog A (VPS4A) or VPS4B mutants, and knockout of γ2-adaptin blocked HBV assembly and egress (Hartmann-Stuhler and Prange, 2001; Rost et al., 2006; Lambert et al., 2007; Figure 1). However, the manipulation of these proteins did not alter the secretion of subviral particles. Also involved in viral egress is Neural precursor cell Expressed, Developmentally Down-regulated 4 (NEDD) E3 ubiquitin protein ligase, which appears to control virus production by binding to the late assembly domain-like PPAY motif of HBV capsids (Rost et al., 2006; Garcia et al., 2013). It is also known that at some point, autophagy is involved in HBV production as the S-protein was shown to interact with the autophagy factor LC3 and manipulations to the pathway result in changes in HBV secretion (Li et al., 2011).

## Concluding Remarks

The interactome we build of the cccDNA, HBc and HBx protein in this review emphasizes the vast amount of knowledge there is about the interactions between HBV proteins and in particular HBx, HBc and the cccDNA. To our knowledge, this is the first time the information has been brought together in a comprehensive overview. Bringing this information together, it shows that there are still clear gaps in knowledge. For example, the network shows that several proteins were only described in a single publication as an interacting protein of cccDNA, HBc, or HBx. Further characterization of this kind of interactions and potentially understanding the reason behind these interactions will greatly benefit the understanding of HBV-related processes. In addition, through analysis of the known interacting proteins, we predicted 210 proteins which potentially interact with either cccDNA, HBx, HBc, or with multiple key modalities of HBV.

Experimental verification of these proteins can lead to the discovery of novel mechanisms and expansion of known protein interaction networks.

Being able to position cccDNA, HBc and HBx in the greater whole of the cellular environment is paramount to better understand how HBV hijacks the cellular environment.

## Author Contributions

EVD, JV, LV, and FP conceived, designed, and wrote the manuscript. BS performed the network pathway analysis, designed the gene association network, and contributed to scientific discussions about the generated network. All authors have read and edited the manuscript.

## Conflict of Interest

EVD, FP, LV, and BS are employees of Janssen Research and Development and may be Johnson & Johnson stockholders. JV was employed at Janssen Research and Development at the time of the work and drafting of the manuscript and may be Johnson & Johnson stockholder.

## Publisher’s Note

All claims expressed in this article are solely those of the authors and do not necessarily represent those of their affiliated organizations, or those of the publisher, the editors and the reviewers. Any product that may be evaluated in this article, or claim that may be made by its manufacturer, is not guaranteed or endorsed by the publisher.
